# Indiana Dental College

**Published:** 1882-05

**Authors:** 


### INDIANA DENTAL COLLEGE.

The third annual commencement.

The number of matriculates for the session was twenty-eight.

The degree of D.D.S. was conferred on the following graduates.

| NAME. | RESIDENCE. | NAME. | RESIDENCE. |
|---|---|---|---|
| J. E. Bodine | Ohio | J. L. Mahan | Ohio. |
| F. W. Blomily | Wisconsin. | D. G. Parker | Louisiana. |
| F. M. Harris | Indiana. | W. M. Ramsdell | New York. |
| L. L. Hinshaw | Indiana. | F. Sawhill | Nebraska. |
| R. E. Henshie | Illinois. | E. W. Sheriff | Colorado. |
| W. F. Kennedy | Indiana. | D. R. Smith | Illinois. |
| C. A. Murray | Ohio. | W. W. Shryock | Indiana. |
| T. R. Woodard | Indiana. | | |

